# Autocrine IFNγ Controls the Regulatory Function of Lymphoproliferative Double Negative T Cells

**DOI:** 10.1371/journal.pone.0047732

**Published:** 2012-10-15

**Authors:** Stephen C. Juvet, Mei Han, Ramesh Vanama, Betty Joe, Edward Y. Kim, Fei Linda Zhao, Caroline Jeon, Oyedele Adeyi, Li Zhang

**Affiliations:** 1 Institute of Medical Science, University of Toronto, Toronto, Ontario, Canada; 2 Division of Respirology and Toronto Lung Transplant Program, Department of Medicine, University Health Network, University of Toronto, Toronto, Ontario, Canada; 3 Clinician-Scientist Training Program, Department of Medicine, University of Toronto, Toronto, Ontario, Canada; 4 Department of Laboratory Medicine and Pathobiology, University of Toronto, Toronto, Ontario, Canada; 5 Department of Immunology, University of Toronto, Toronto, Ontario, Canada; 6 Toronto General Research Institute, University Health Network, Toronto, Ontario, Canada; National Cancer Institute (INCA), Brazil

## Abstract

TCRαβ^+^ CD4^−^CD8^−^NK^−^ double negative T cells (DN T cells) can act as regulatory T cells to inhibit allograft rejection and autoimmunity. Their role in graft-versus-host disease and mechanisms of suppression remain elusive. In this study, we demonstrate that DN T cells can inhibit CD4^+^ T cell-mediated GVHD in a semi-allogeneic model of bone marrow transplantation. Furthermore, we present evidence of a novel autocrine IFNγ signaling pathway in Fas-deficient C57BL/6.*lpr* (B6.*lpr*) DN T cells. B6.*lpr* DN T cells lacking IFNγ or its receptor were impaired in their ability to suppress syngeneic CD4^+^ T cells responding to alloantigen stimulation both *in vitro* and *in vivo*. Autocrine IFNγ signaling was required for sustained B6.*lpr* DN T cell IFNγ secretion *in vivo* and for upregulation of surface Fas ligand expression during TCR stimulation. Fas ligand (FasL) expression by B6.*lpr* DN T cells permitted lysis of activated CD4^+^ T cells and was required for suppression of GVHD. Collectively, our data indicate that DN T cells can inhibit GVHD and that IFNγ plays a critical autocrine role in controlling the regulatory function of B6.*lpr* DN T cells.

## Introduction

DN T cells express an αβ T cell receptor (TCR) but do not express either CD4 or CD8 coreceptors, nor do they express NK cell markers. This phenotype differentiates them from other unconventional T cells (e.g. NK T cells and γδ T cells). Depending on the context, DN T cells have been shown to have regulatory, innate, or pathogenic properties [1]. In murine models, DN T cells can act as regulatory T cells (Tregs) that inhibit allo- and xenograft rejection [2], [3], [4], [5] and autoimmune diabetes [6], [7]. Our previous studies showed that TCR transgenic DN T cells attenuated CD8^+^ T cell-induced graft-versus-host disease (GVHD) in a single class I-mismatched mouse model [8]. Whether DN T cells can suppress CD4^+^ T cell-mediated GVHD is not known. Human DN T cells inhibit autologous CD4^+^ and CD8^+^ T cell proliferation *in vitro* [9], [10], and the frequency of circulating DN T cells inversely correlated with the severity of GVHD in bone marrow transplantation (BMT) patients, suggesting that DN T cells might inhibit GVHD in humans [11].

Fas or FasL mutations in mice (*lpr* and *gld* respectively) and humans (autoimmune lymphoproliferative syndrome) exhibit lymphoproliferation, autoimmunity, and DN T cell expansion. Whereas some studies implicate DN T cells in the pathogenesis of autoimmunity in these settings, *lpr* DN T cells can also act as Tregs in some contexts [12], [13], for example following an infusion of allogeneic lymphocytes [12]. This feature is shared with other murine [2], [14] and human [10] DN T cells. Furthermore FasL-expressing T cells in NOD.*lpr* mice can resist diabetes induced by adoptively transferred T cells [15]. Whether *lpr* DN T cells can inhibit syngeneic CD4^+^ T cell responses is of interest since the latter are autoimmune effectors in *lpr* and *gld* mice [16], [17].

IFNγ, whose inflammatory role is well described, is also immunoregulatory: it helps clear activated T cells [18], induces Foxp3^+^ Tregs [19], inhibits IL-17-secreting T cells [20], and upregulates immunoregulatory enzymes in antigen presenting cells (APCs) [21], [22]. IFNγ is expressed by mouse [2], [23], rat [24] and human [25] DN T cells but its role in DN T cell function and the underlying mechanisms are not clear.

Here, we demonstrate that alloantigen-primed BALB/c and Fas-deficient B6.*lpr* DN T cells can act as Tregs to inhibit GVHD mediated by syngeneic CD4^+^ T cells in a semiallogeneic BMT model. Furthermore, we have identified a novel IFNγ-dependent autocrine mechanism that is critical for B6.*lpr* DN T cell-mediated immune suppression *in vitro* and *in vivo*.

## Results

### DN T cells can inhibit CD4^+^ T cell-mediated GVHD

**Figure 1:**
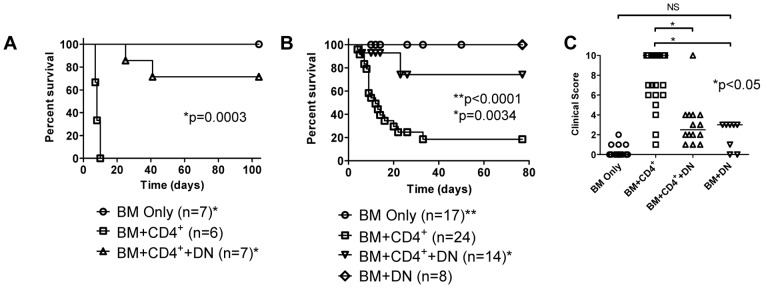
DN T cells inhibit semi-allogeneic CD4^+^ T cell-induced GVHD. **A.** Lethally irradiated CB6F1 mice were reconstituted with BALB/c TCD BM alone (BM only, n = 7) or with BALB/c CD4^+^ T cells, without (BM+CD4^+^, n = 6) or with BALB/c DN-enriched lymphocytes (BM+CD4^+^+DN, n = 7). A clinical score [26] incorporating posture, fur texture, activity level, skin integrity and weight loss was assigned 3 times weekly and survival was monitored daily. Data from 3 independent experiments (each with 2–3 mice per group) are shown; *log rank p = 0.0003 vs. BM+CD4^+^. **B.** CB6F1 recipients conditioned as in A were reconstituted with B6 TCD BM alone (BM only) or with B6.Thy1.1 CD4^+^ T cells, without (BM+CD4^+^) or with B6.*lpr* DN T cells (BM+CD4^+^+DN). Data are from 5 independent experiments, each with up to 6 mice per group; *log rank p = 0.0034 vs. BM+CD4^+^; **log rank p<0.0001 vs. BM+CD4^+^. **C.** Clinical score was determined 2 weeks after BMT in recipients of BM only (n = 17), BM+CD4^+^ (n = 24), BM+CD4^+^+DN (n = 14) and BM+DN (n = 8). Data are derived from 5 independent experiments, each with up to 6 mice per group. Kruskal-Wallis test p<0.0001; *Dunns post test p<0.05.

To date, whether non-transgenic, polyclonal DN T cells can inhibit GVHD is unknown. To address this question, lethally irradiated (B6xBALBc)F1 (CB6F1) mice (H-2^b/d^) were transplanted with T cell-depleted (TCD) BALB/c (H-2^d^) bone marrow cells alone (BM only) or with BALB/c CD4^+^ T cells, without (BM+CD4^+^) or with preactivated and enriched (by depleting B cells and CD8^+^ and CD4^+^ T cells) BALB/c DN T cells (BM+CD4^+^+DN) as putative suppressor cells. GVHD clinical score [26] and recipient survival were monitored daily. Mice receiving BM alone had 100% long-term survival, while mice receiving BM+CD4^+^ succumbed to acute GVHD (median survival 8 days, **Fig. 1A**, p = 0.0003). In contrast, the CB6F1 mice treated with alloantigen-primed BALB/c cells enriched for DN T cells showed markedly reduced GVHD (5 of 7 mice survived >100 days, **Fig. 1A**, p = 0.0003).

Due to a very low frequency of DN T cells in normal mice, we were unable to obtain sufficient numbers of DN T cells with high purity from BALB/c mice to confirm that these results were exclusively attributable to DN T cells. On the other hand, DN T cells are greatly expanded in Fas-deficient *lpr* mice and can exhibit regulatory function [12], [13], although they are generally regarded as pathogenic autoimmune effector cells within the *lpr* context [27], [28]. Whether they might inhibit GVHD, or in fact worsen the disease, is not known. Intriguingly, unlike allogeneic CD4^+^ or CD8^+^ T cells which cause severe GVHD [29], infusion of purified (**Fig. S1**) B6.*lpr* DN T cells pre-activated by alloantigen *in vivo* did not cause significant illness or mortality in lethally irradiated CB6F1 mice reconstituted with B6 (H-2^b^) BM (BM+DN, **Fig. 1B–C**). These mice experienced a mild, transient illness (clinical score ≤3, median survival >80d). To confirm that DN T cells are able to inhibit allogeneic CD4^+^ T cell-induced GVHD, we purified preactivated B6.*lpr* DN T cells and administered them to CB6F1 recipients of B6 BM and B6. Thy1.1 CD4^+^ T cells. Whereas recipients of BM only had no GVHD and all survived >80d (**Fig. 1B**) mice receiving BM+CD4^+^ developed acute GVHD (median survival 12d, p<0.0001). Importantly, infusion of preactivated B6.*lpr* DN T cells increased median survival from 12 to >80 days (log rank test; p = 0.0034), and decreased illness severity compared with BM+CD4^+^ treated mice (**Fig. 1B–C**). These data demonstrate that infusion of allogeneic B6.*lpr* DN T cells does not cause severe illness and can prevent death in mice undergoing CD4^+^ T cell-mediated GVHD.

**Figure 2:**
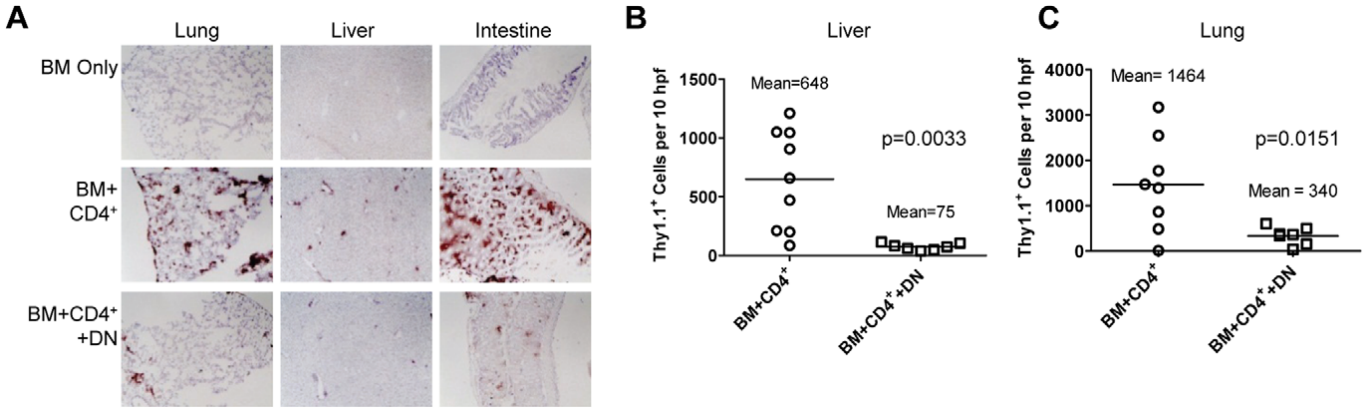
B6.lpr DN T cells decrease GVHD target organ infiltration by alloreactive CD4^+^ T cells. Lethally irradiated CB6F1 recipients were reconstituted with B6 TCD BM alone (BM only) or with B6. Thy1.1 CD4^+^ T cells, without (BM+CD4^+^) or with B6.*lpr* DN T cells (BM+CD4^+^+DN). Major GVHD target organs were harvested at two weeks after BMT and infiltrating donor T cells were identified by staining frozen tissue sections with anti-Thy1.1 mAb. **A.** Representative (3–9 mice per group) 4× photomicrographs of lung (left column), liver (middle panels), and intestine (right panels) sections are shown. The number of Thy1.1^+^ cells in 10 high power fields (40× objective) was reduced in mice receiving B6.*lpr* DN T cells (n = 7) compared with mice not receiving B6.*lpr* DN T cells (n = 9) in liver (**B**, t test p = 0.0033) and lung (**C**, t test p = 0.0151).

Furthermore, GVHD protection by B6.*lpr* DN T cells was associated with decreased lung, liver, and intestinal infiltration by CD4^+^ T cells (**Fig. 2A** **, left**, **middle**, and **right columns**, respectively). Effector cell density in the intestine precluded accurate quantification, but we observed statistically significant reductions in alloreactive T cell numbers in the liver (p = 0.0033, **Fig. 2B**) and lungs (p = 0.0151, **Fig. 2C**) when B6.*lpr* DN T cells were included in the inoculum. Taken together, these data suggest that B6.*lpr* DN T cells prevent GVHD mortality by reducing CD4^+^ T cell infiltration of GVHD target tissues, thereby reducing organ injury.

### IFNγ is critical for B6.lpr DN T cell-mediated suppression of syngeneic T cell responses to alloantigen

**Figure 3:**
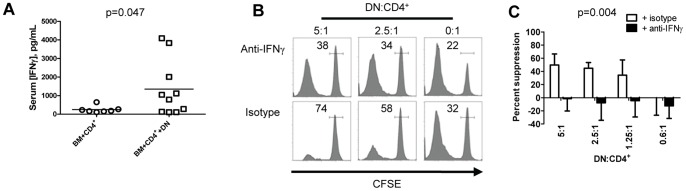
IFNγ is involved in B6.lpr DN T cell regulatory function. **A.** Lethally irradiated CB6F1 recipients were reconstituted with B6 TCD BM alone (BM only) or with B6. Thy1.1 CD4^+^ T cells, without (BM+CD4^+^) or with B6.*lpr* DN T cells (BM+CD4^+^+DN). Five days after BMT, serum IFNγ was elevated in BM+CD4^+^+B6.*lpr* DN-treated mice (n = 10) compared to BM+CD4^+^-treated mice (n = 7). Unpaired t test with Welch's correction, p = 0.047. **B.** CFSE-labelled B6. Thy1.1 CD4^+^ T cells were cultured with IL-2, irradiated CB6F1 splenocytes and B6.*lpr* DN T cells in indicated ratios, with anti-IFNγ mAb or isotype control. CFSE dilution in live Thy1.1^+^CD4^+^ cells was determined after 5 days. CFSE histograms from one of three independent experiments are shown. Numbers inside histograms reflect the percentage of cells in the CFSE^hi^ gate. **C.** Compiled data from three independent experiments shown. Two-way ANOVA p = 0.004.

IFNγ can contribute to GVHD pathogenesis; however, early IFNγ administration can inhibit GVHD [30], [31]. DN T cells from humans [9], TCR transgenic mice [2], rats [24] and *lpr* mice [23] express IFNγ. To ascertain whether B6.*lpr* DN T cells might cause an early rise in IFNγ during GVHD, serum IFNγ levels were determined by ELISA 5 days after BMT. Compared with recipients of BM+CD4^+^, recipients of BM+CD4^+^+DN had elevated serum IFNγ (**Fig. 3A** **,** p = 0.047), suggesting that IFNγ might contribute to B6.*lpr* DN T cell-mediated suppression of GVHD.

To determine the importance of IFNγ in B6.*lpr* DN T cell regulatory function, CFSE labelled Thy1.1^+^CD4^+^ T cells were co-cultured with B6.*lpr* DN T cells, with either neutralizing anti-IFNγ mAb or isotype control IgG. IFNγ neutralization slightly increased the proportion of proliferated CD4^+^ T cells in the absence of B6.*lpr* DN T cells (**Fig. 3B** **, right histograms**). Importantly, IFNγ neutralization markedly hampered the function of B6.*lpr* DN T cells (**Fig. 3B–C**).

**Figure 4:**
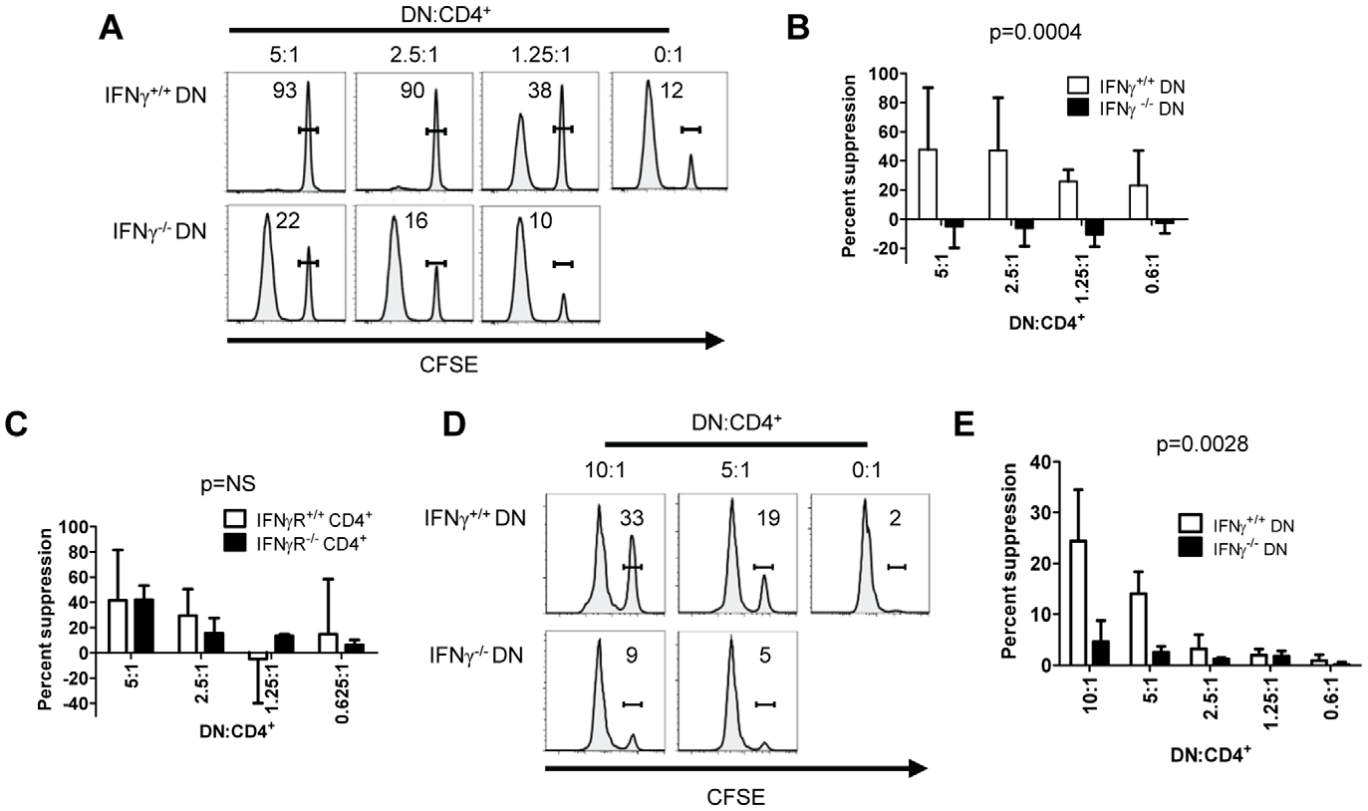
B6.lpr DN T cell- secreted IFNγ is required, but APCs are not, for inhibition of alloreactive CD4^+^ T cell proliferation in vitro. **A.** In varying ratios, B6.*lpr*.IFNγ^−/−^ or B6.*lpr*.IFNγ^+/+^ DN T cells were cultured with CFSE-labelled B6. Thy1.1 CD4^+^ T cells in the same conditions as Fig. 3B for 5 days. CFSE histograms, gated on Thy1.1^+^CD4^+^ cells, from one of three independent experiments are shown. Numbers inside histograms reflect the percentage of cells in the CFSE^hi^ gate. **B.** Compiled data from three independent experiments are shown. Two-way ANOVA p = 0.0004. **C.** In varying ratios, B6.*lpr* DN T cells were cultured with CFSE-labelled B6. Thy1.1.IFNγR^−/−^ or B6. Thy1.1.IFNγR^+/+^ CD4^+^ T cells in the same conditions as Fig. 3B for 5 days. Two-way ANOVA p = 0.93. **D.** In varying ratios, B6.*lpr*.IFNγ^−/−^ or B6.*lpr*.IFNγ^+/+^ DN T cells were cultured with CFSE-labelled B6. Thy1.1 CD4^+^ T cells for 3 days with soluble anti-CD28 mAb and plate-bound anti-CD3 mAb. CFSE histograms from one of two independent experiments with similar results are shown. **E.** Data from two independent experiments are shown; two-way ANOVA p = 0.0028.

To confirm the importance of DN T cell-derived IFNγ, we generated B6.*lpr*.IFNγ^−/−^ mice and assessed the ability of their DN T cells to suppress CD4^+^ T cell proliferation. Although B6.*lpr*.IFNγ^−/−^ DN T cells exerted a slight inhibition of CD4^+^ T cell proliferation at a 5∶1 DN:CD4^+^ ratio (**Fig. 4A** **, bottom left histogram**), they did not suppress this response efficiently compared with B6.*lpr*.IFNγ^+/+^ DN T cells (**Fig. 4B**, p = 0.0004), indicating that IFNγ secretion by B6.*lpr* DN T cells is involved in their inhibitory function *in vitro*.

IFNγ promotes activation-induced cell death of T lymphocytes [32]. To examine whether DN T cell-derived IFNγ might act on CD4^+^ T cells, we generated IFNγ receptor 1-deficient B6. Thy1.1.IFNγR^−/−^ mice and compared the proliferation of their CD4^+^ T cells with that of B6. Thy1.1.IFNγR^+/+^ CD4^+^ T cells in the presence of B6.*lpr* DN T cells. Surprisingly, both types of CD4^+^ T cell were suppressed equally by B6.*lpr* DN T cells (p = 0.93, **Fig. 4C**), suggesting that inhibition of CD4^+^ T cell proliferation by B6.*lpr* DN T cells is not mediated by a direct effect of IFNγ on the former. Furthermore, we stimulated CFSE-labelled B6. Thy1.1 CD4^+^ T cells to proliferate using plate-bound anti-CD3 and soluble anti-CD28 antibodies. In this setting, B6.*lpr* DN T cells, but not B6.*lpr*.IFNγ^−/−^ DN T cells, efficiently inhibited T cell proliferation (p = 0.0028, **Fig.** **4D–E**). This observation indicates that B6.*lpr* DN T cells can inhibit the proliferative response of syngeneic CD4^+^ T cells in an IFNγ-dependent fashion, but that the B6.*lpr* DN T cell-derived IFNγ does not act on APCs to mediate this effect.

### B6.lpr DN T cell IFNγ secretion and signaling are required for suppression of GVHD

**Figure 5:**
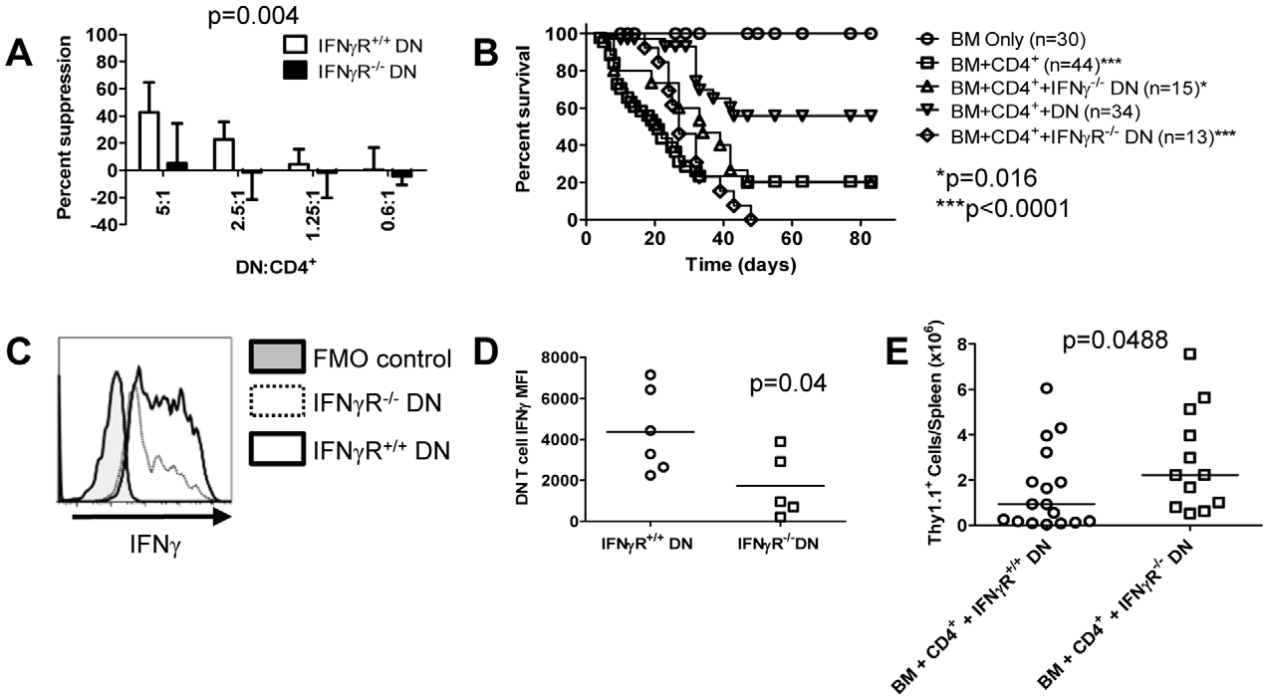
B6.lpr DN T cell IFNγR expression is critical for their regulatory function. A. In varying ratios, B6.*lpr*.IFNγR^−/−^ or B6.*lpr*.IFNγR^+/+^ DN T cells were cultured with CFSE-labelled B6. Thy1.1.IFNγR^−/−^ CD4^+^ T cells and irradiated CB6F1.IFNγR^−/−^ splenocytes and IL-2 for 5 days. Suppression of Thy1.1 CD4^+^ T cell proliferation by B6.*lpr*.IFNγR^−/−^ or B6.*lpr*.IFNγR^+/+^ DN T cells was determined by flow cytometry. Data from 6 independent experiments are shown; two-way ANOVA p = 0.004. **B.** Lethally irradiated CB6F1 mice were reconstituted with BM only (n = 30), BM+CD4^+^ (n = 44), or BM+CD4^+^ plus B6.*lpr* DN (n = 34), B6.*lpr*.IFNγ^−/−^ DN (n = 15), or B6.*lpr*.IFNγR^−/−^ DN (n = 13). Recipients were monitored for clinical score and survival as in Fig. 1. Data from Fig. 1B (5 experiments) are included plus 4 additional independent experiments, each with 2–6 mice per group. In comparison with BM+CD4^+^+B6.*lpr* DN: *log rank p = 0.016 (B6.*lpr* IFNγ^−/−^ DN); ***log rank p<0.0001 (B6.*lpr*.IFNγR^−/−^ DN); ***log rank p<0.0001 (BM+CD4^+^). **C.** Representative IFNγ histograms, gated on DN T cells, from one of 6 recipients of BM+CD4^+^+B6.*lpr*.IFNγR^+/+^ DN (solid line) and one of 5 recipients of BM+CD4^+^+B6.*lpr*.IFNγR^−/−^ DN are compared with fluorescence-minus-one control (shaded). **D.** Compiled data from all 11 mice in C are shown. Horizontal lines represent mean MFI; t test p = 0.043. **E.** Two weeks after BMT, a lower number of Thy1.1^+^ cells were recovered from the spleens of mice treated with BM+CD4^+^+B6.*lpr* DN T cells as compared with BM+CD4^+^+B6.*lpr*.IFNγR^−/−^ DN T cells. Data from 17 recipients of BM+CD4^+^+B6.*lpr*.IFNγR^+/+^ DN T cells and 12 recipients of BM+CD4^+^+B6.*lpr*.IFNγR^−/−^ DN T cells pooled from three independent experiments are shown.

We next tested whether IFNγ signalling might underlie B6.*lpr* DN T cell function. To this end, we generated B6.*lpr*.IFNγR^−/−^ mice, and tested the ability of their DN T cells to suppress CD4^+^ T cells *in vitro*. To identify the specific contribution of B6.*lpr* DN T cell IFNγ signalling, CFSE-labelled B6. Thy1.1.IFNγR^−/−^ CD4^+^ T cells were stimulated by CB6F1.IFNγR^−/−^ splenocytes and their proliferation was measured by CFSE dilution. As shown in **Fig. 5A**, B6.*lpr*.IFNγR^−/−^ DN T cells exhibited a decreased ability to inhibit CD4^+^ T cell proliferation compared with B6.*lpr*.IFNγR^+/+^ DN T cells (p = 0.004).

To determine whether B6.*lpr* DN T cells lacking either IFNγ or its receptor differ in their ability to inhibit GVHD, lethally irradiated CB6F1 recipients were reconstituted with BM alone, BM+CD4^+^, or BM+CD4^+^+ B6.*lpr*, B6.*lpr*.IFNγR^−/−^ or B6.*lpr*.IFNγ^−/−^ DN T cells. Consistent with our *in vitro* data, mice receiving either IFNγ^−/−^ or IFNγR^−/−^ B6.*lpr* DN T cells experienced higher GVHD mortality than mice receiving IFNγ^+/+^ and IFNγR^+/+^ B6.*lpr* DN T cells, with median survival times of 34 days (B6.*lpr*.IFNγ^−/−^ DN, p = 0.016) and 27 days (B6.*lpr*.IFNγR^−/−^ DN, p<0.0001) compared with >80 days (B6.*lpr* DN, **Fig. 5B**). Taken together, these findings reveal that B6.*lpr* DN T cells must both secrete and respond to IFNγ in order to exert regulatory function *in vitro* and *in vivo*.

These observations prompted us to examine for evidence of an autocrine relationship between IFNγ secretion and signaling in B6.*lpr* DN T cells. Two weeks after BMT, splenocytes from CB6F1 recipients of BM+CD4^+^+B6.*lpr*.IFNγR^−/−^ or B6.*lpr*.IFNγR^+/+^ DN T cells were restimulated *in vitro* and IFNγ expression in the DN T cell compartment was examined. Consistent with an autocrine process, B6.*lpr*.IFNγR^−/−^ DN T cells failed to synthesize as much IFNγ as B6.*lpr* DN T cells as determined by flow cytometry (median fluorescence intensity 1737±710 vs. 4370±830, p = 0.04, **Fig. 5C–D**).

We next assessed whether the absence of IFNγ signaling in B6.*lpr* DN T cells might be associated with an impaired ability to suppress donor T cells in the spleen. We observed a significantly lower number of Thy1.1^+^ CD4^+^ cells in mice receiving B6.*lpr*.IFNγR^+/+^ vs. B6.*lpr*.IFNγR^−/−^ DN T cells (p = 0.0488, **Fig. 5E**). Taken together, these data indicate that in B6.*lpr* DN T cells unable to respond to IFNγ, IFNγ synthesis in response to TCR stimulation is profoundly impaired, and that this impairment is associated with a decreased ability to inhibit the expansion of alloreactive T cells *in vivo* during GVHD.

### Autocrine IFNγ signaling increases surface FasL expression by B6.lpr DN T cells

**Figure 6:**
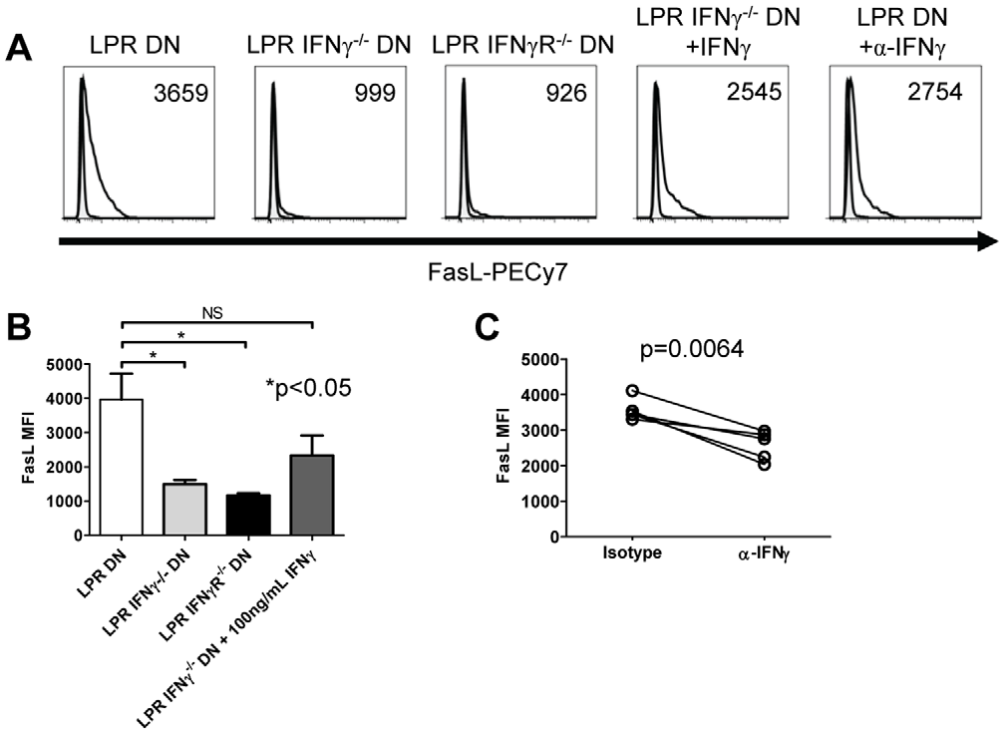
Autocrine IFNγ controls FasL surface expression by B6.lpr DN T cells. **A.** B6.*lpr* (first panel), B6.*lpr*.IFNγ^−/−^ (second panel) and B6.*lpr*.IFNγR^−/−^ (third panel) spleen and lymph node cells were cultured on plate-bound anti-CD3 with 5 µg/mL soluble anti-CD28 and IL-2 for 48h. B6.*lpr*.IFNγ^−/−^ cells were also given 100ng/mL IFNγ (fourth panel), and B6.*lpr* DN T cells were also treated with anti-IFNγ mAb (fifth panel) or isotype control (not shown). Representative examples of surface FasL expression (gated on DN T cells) are shown in comparison with fluorescence-minus-one controls (shaded). Numbers in histograms reflect MFI. **B.** Compiled data from 12 B6.*lpr*, 12 B6.*lpr*.IFNγ^−/−^, and 6 B6.*lpr*.IFNγR^−/−^ mice in 6 separate experiments are shown. One-way ANOVA p = 0.0098; *Bonferroni post test p<0.05 **C.** B6.*lpr* DN T cells were activated as in A for 48h with either IFNγ neutralizing antibody or isotype control. Data from 2 separate experiments with 5 individual mice are shown. Paired t test p = 0.0064.

Our previous work revealed that syngeneic Fas-sufficient T cells are suppressed by activated B6.*lpr* DN T cells at least partially via direct FasL-mediated cytotoxicity [12]. To determine whether IFNγ might promote surface expression of FasL in B6.*lpr* DN T cells as shown in human CD4^+^ T cells [33], B6.*lpr*, B6.*lpr*.IFNγ^−/−^, and B6.*lpr*.IFNγR^−/−^ lymphocytes were activated and examined for FasL expression. Activated B6.*lpr* DN T cells upregulated surface FasL over 48h (**Fig. 6A** **, first panel** and **Fig. 6B**) while B6.*lpr*.IFNγ^−/−^ and B6.*lpr*.IFNγR^−/−^ DN T cells did not (**Fig. 6A** **, second and third panels** and **Fig. 6B**). Importantly, all 3 types of DN T cells expressed similar levels of CD69 and CD25 as a result of this stimulation (**data not shown**). Consistent with a role for IFNγ signalling, supplementation of B6.*lpr*.IFNγ^−/−^ DN T cell cultures with IFNγ partially restored surface FasL expression, although not to levels seen in IFNγ-sufficient B6.*lpr* DN T cells (**Fig. 6A** **, fourth panel** and **Fig. 6B**). Similarly, neutralization of IFNγ during activation of B6.*lpr* DN T cells reduced surface FasL expression (**Fig. 6A** **, fifth panel** and **Fig. 6C**). The observed differences in FasL expression were not due to altered proteolytic processing of FasL, as the addition of the metalloproteinase inhibitor TAPI-1 during the final 18h of culture resulted in slight increases in FasL expression in all three types of DN T cells (**Fig. S2**). Interestingly, all three types of DN T cells had similar intracellular FasL expression (**data not shown**), consistent with the notion that IFNγ does not influence FasL protein synthesis in B6.*lpr* DN T cells, but promotes its cell surface expression.

**Figure 7:**
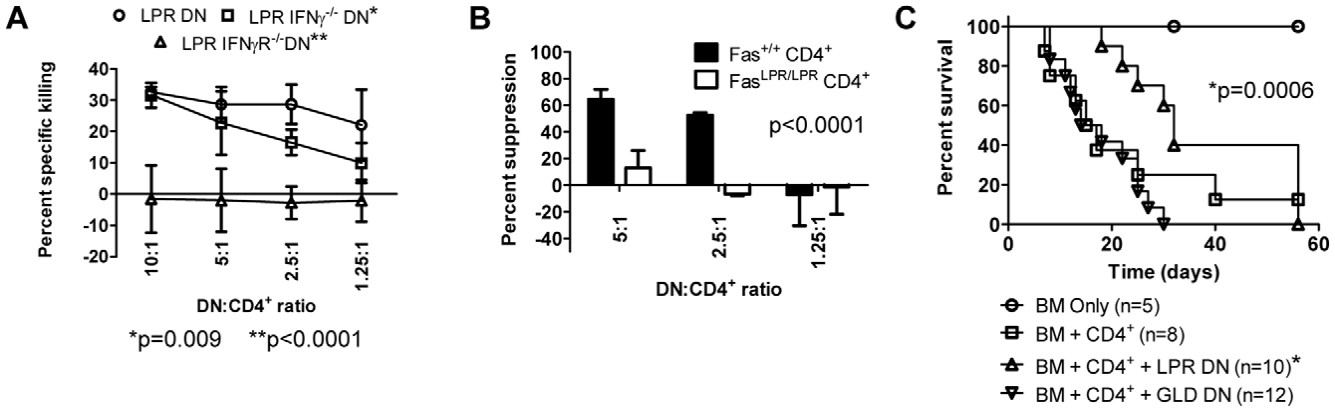
IFNγ secretion and signalling and FasL expression enable B6.lpr DN T cells to inhibit alloreactive CD4^+^ T cells in vitro and in vivo. **A.** Preactivated ^3^H-thymidine labelled CD4^+^ T cells were cultured for 18h with preactivated B6.*lpr*, B6.*lpr*.IFNγ^−/−^, or B6.*lpr*.IFNγR^−/−^ DN T cells at indicated ratios. Specific cytotoxicty was determined based on retention of ^3^H-thymidine in viable targets. Data are compiled from 4 experiments in which, respectively, B6.*lpr* (circles), B6.*lpr*. IFNγ^−/−^ (squares), or B6.*lpr*.IFNγR^−/−^ (triangles) DN T cells were used in 4, 3, and 2 experiments. Compared with B6.*lpr* DN, *Two-way ANOVA p = 0.0009 and **p<0.0001. **B.** In varying ratios, B6.*lpr* DN T cells were cultured with CFSE-labelled B6. Thy1.1 (Fas^+/+^) or B6.*lpr* (Fas*^lpr/lpr^*) CD4^+^ T cells, irradiated CB6F1 splenocytes and IL-2. CFSE dilution in live (7-AAD^−^) cells was determined after 5 days. Data from 2 independent experiments are shown; two-way ANOVA p<0.0001. **C.** Lethally irradiated CB6F1 mice received BM only (n = 5), BM+CD4^+^ (n = 8), BM+CD4^+^+2.5×10^6^ B6.*lpr* DN (n = 10, FasL^+^) or 2.5×10^6^ B6.*gld* DN (n = 12, FasL^−^). Data are from 2 independent experiments, each with 2–6 mice per group. *Log rank p = 0.006 vs. B6.*gld* DN.

To investigate whether autocrine IFNγ signalling is involved in the cytotoxic function of these cells, activated B6.*lpr*, B6.*lpr*.IFNγ^−/−^, and B6.*lpr*.IFNγR^−/−^ DN T cells were used as effectors to kill preactivated B6 CD4^+^ T cells. While B6.*lpr* DN T cells efficiently killed the targets, B6.*lpr*.IFNγ^−/−^ DN T cells exhibited reduced cytotoxicity (p = 0.009, **Fig. 7A**). B6.*lpr*.IFNγR^−/−^ DN T cells, which do not express FasL, were not able to kill activated syngeneic CD4^+^ T cells (p<0.0001, **Fig. 7A**). These data support the hypothesis that autocrine IFNγ is required for killing of activated syngeneic CD4^+^ T cells by B6.*lpr* DN T cells. Consistent with these findings, whereas B6.*lpr* DN T cells suppressed Fas-sufficient B6.Thy1.1 CD4^+^ T cells, they were unable to suppress Fas-deficient B6.*lpr* CD4^+^ T cells effectively (**Fig. 7B**, p<0.0001). Furthermore, using 7AAD to identify dead cells, we observed a dose-dependent increase in cell death among proliferated B6. Thy1.1 CD4^+^ T cells, but not among B6.*lpr* CD4^+^ T cells (**Fig. S3**).

Finally, to confirm the importance of B6.*lpr* DN T cell FasL expression in GVHD suppression, lethally irradiated CB6F1 mice were transplanted with BM+CD4^+^ and either B6.*lpr* (FasL^+^) or B6.*gld* (lacking functional FasL) DN T cells. In these experiments, a dose of 2.5×10^6^, rather than 5×10^6^, DN T cells was given to each recipient. Despite this lower dose, B6.*lpr* DN T cells significantly enhanced GVHD survival compared with B6.*gld* DN T cells (**Fig. 7C**; log rank test p = 0.0006), whereas B6.*gld* DN T cell-treated mice had a rate of survival comparable to mice treated with BM+CD4^+^ (**Fig. 7C**).

Collectively these data reveal that B6.*lpr* DN T cell regulatory function is dependent on autocrine IFNγ signaling and on expression of a functional FasL molecule *in vitro* and during GVHD *in vivo*.

## Discussion

In this study, we demonstrated that alloantigen-primed DN T cells can inhibit CD4^+^ T cell-induced GVHD in a semi-allogeneic mouse model. DN T cells from humans and rodents synthesize IFNγ [2], [23], [24], [25], but its role in DN T cell function has been unclear. Here we have shown that IFNγ secretion and signaling in B6.*lpr* DN T cells is required for these cells to act as Tregs toward alloreactive CD4^+^ T cells *in vitro* and *in vivo*. Since B6.*lpr* DN T cells were able to suppress proliferation of CD4^+^ T cells from both B6. Thy1.1.IFNγR^−/−^ and B6. Thy1.1.IFNγR^+/+^ mice (**Fig. 4C**), and did not require APCs for this effect *in vitro* (**Fig. 4D–E**), our findings suggested that IFNγ likely acts in an autocrine fashion in B6.*lpr* DN T cells. Although our data do not exclude a role for B6.*lpr* DN T cell-derived IFNγ acting on APCs *in vivo*, the following findings further support a role for IFNγ autocrine signalling in the regulatory function of these cells *in vivo*: 1) B6.*lpr*.IFNγR^−/−^ DN T cells failed to synthesize significant IFNγ *in vivo* (**Fig. 5C–D**); 2) expression of both IFNγ and its receptor by B6.*lpr* DN T cells was important for B6.*lpr* DN T cell-mediated suppression of GVHD (**Fig. 5B** and **Fig. 5E**); and 3) both IFNγ secretion and signaling were required for B6.*lpr* DN T cell surface FasL expression (**Fig. 6**), which was also required for GVHD inhibition (**Fig. 7C**). Collectively, these data support the notion that B6.*lpr* DN T cells are required to release IFNγ and respond to this cytokine by upregulating surface FasL upon TCR stimulation, in order to inhibit alloimmune responses by syngeneic Fas-sufficient CD4^+^ T cells. We must concede, however, that our data do not definitively demonstrate that IFNγ-mediated FasL surface expression in B6.*lpr* DN T cells is the mechanism by which CD4^+^ T cells were inhibited during GVHD. Confirmation of this phenomenon would require an approach such as inhibition of FasL expression in IFNγ- and IFNγR-sufficient B6.*lpr* DN T cells, or alternatively, rescue of the regulatory function of IFNγR-deficient B6.*lpr* DN T cells by overexpression of FasL in these cells.

Hill and colleagues induced cardiac allograft tolerance by treating rats with autologous immature DCs and LF15-0195 [24]. Interestingly, IFNγ secretion by splenic DN T cells was required for tolerance, and IFNγ neutralization *in vivo* resulted in allograft rejection. IFNγ^+^ cells in the spleen were visualized in contact with apoptotic T cells [24]. These findings demonstrate a regulatory role for DN T cell-secreted IFNγ in a different species and suggest that it is involved in the deletion of effector T cells. However, the mechanism by which IFNγ was acting in that model was not determined. Our data reveal that in B6.*lpr* DN T cells, IFNγ acts in an autocrine fashion and up-regulates surface expression of the key effector molecule FasL. Whether a similar process occurs in DN T cells from other mouse strains or species requires further investigation.

Our findings are also in keeping with recent studies on the role of IFNγ in CD4^+^ Foxp3^+^ Tregs. The latter must release IFNγ shortly after alloantigen encounter to prevent allograft rejection [34]. Wei and colleagues [35] showed that Foxp3^+^ Tregs lacking either IFNγ or IFNγR could not prevent allograft rejection, accompanied by reduced STAT1 activation in these cells. Hence, a similar autocrine IFNγ pathway appears to be operating in alloantigen-reactive Foxp3^+^ Tregs.

We have demonstrated that autocrine IFNγ upregulates B6.*lpr* DN T cell surface expression of FasL; the latter is required for their ability to kill activated T cells and suppress GVHD (**Fig. 6** **–** **7** and **Fig. S3**). Only B6.*lpr* DN T cells capable of secreting and responding to IFNγ were able to express high levels of surface FasL (**Fig. 6A**). Furthermore, inhibition of metalloproteinase activity resulted in only a slight increase in FasL expression in B6.*lpr* DN T cells regardless of their ability to synthesize or respond to IFNγ (**Fig. S2**). These data are consistent with a previous report that FasL is stored within secretory lysosomes in T cells, and subsequently externalized upon activation [36]. Our findings therefore suggest that in B6.*lpr* DN T cells, TCR stimulation triggers IFNγ secretion, resulting in autocrine IFNγ signaling and thus externalization of pre-synthesized FasL.

Interestingly, cytotoxicity of B6.*lpr* DN T cells toward activated CD4^+^ T cells was only mildly impaired by IFNγ deficiency but completely abrogated by IFNγR deficiency (**Fig. 7A**). Since antigen re-encounter by previously primed effector T cells results in IFNγ secretion [37], we speculate that IFNγ may be provided partially by the activated CD4^+^ cells in the same culture. B6.*lpr*.IFNγR^−/−^ DN T cells, unable to respond to IFNγ, would thus not be able to externalize FasL. This failure to upregulate surface FasL presumably explains their lack of cytotoxic effect.

Deletion of IFNγ in MRL.*lpr* mice resulted in reduced autoimmunity, lymphoproliferation and mortality [38]. Indeed, IFNγ has been shown to be involved in glomerulonephritis and other autoimmune phenomena in this strain [39], [40]. In contrast, *lpr* mice with other strain backgrounds, including C57BL/6, exhibit lymphoproliferation but only mild autoimmune manifestations in comparison with the MRL background [41], [42]. We have shown here that autocrine IFNγ regulates FasL surface expression in B6.*lpr* DN T cells; as such, owing to the lack of Fas, deletion of IFNγ or its receptor would not be predicted to influence lymphoproliferation in B6.*lpr* mice. Further, owing the fact that autoimmunity is already mild or absent in this strain background, an effect of IFNγ deletion on this aspect of the *lpr* phenotype would be difficult to detect. Indeed, we observed no differences in the longevity or lymphocyte counts of age-matched B6.*lpr*, B6.*lpr*.IFNγ^−/−^, and B6.*lpr*.IFNγR^−/−^ mice (**data not shown**).

A further important observation made in this study is that alloantigen-primed B6.*lpr* DN T cells caused only a very mild illness when infused into lethally irradiated allogeneic recipients (**Fig. 1B–C**). In contrast, transfer of anti-CD3 and IL-23-activated B6.*lpr* lymphocytes into syngeneic RAG1^−/−^ mice produced severe glomerulonephritis [28], and DN T cells were shown to produce larger amounts of IL-17 than other MRL.*lpr* T cell subsets [28]. Moreover, DN T cells have been shown to provide help to B cells in human lupus [43]. Hence, there is strong evidence to suggest that DN T cells contribute to the pathogenesis of lupus-like disease in *lpr* mice. Our data therefore support the premise that the DN T cell compartment of *lpr* mice contains the potential for diverse immunological functions, depending on the context. Whether these diverse functions are attributable to discrete, as-yet unidentified subsets within this compartment, remains to be determined.

Taken together, our data demonstrate that alloantigen-primed DN T cells can inhibit allogeneic CD4^+^ T cell-induced GVHD in mice, and that IFNγ plays a critical autocrine role in controlling the regulatory function of B6.*lpr* DN T cells. Autocrine IFNγ signaling is important for sustained B6.*lpr* DN T cell IFNγ secretion and for upregulation of surface Fas ligand expression, which in turn leads to killing of activated anti-host Fas^+^ CD4^+^ T cells. The role of DN T cell-secreted IFNγ in Fas- and FasL-deficient autoimmune lymphoproliferative states is unclear, and requires further investigation.

## Materials and Methods

### Ethics Statement

Animals were housed in the Toronto Medical Discovery Tower under specific pathogen-free conditions. The animal use protocol (#741) was approved by the University Health Network Animal Care Committee. Animal care was conducted in accordance with the policies and guidelines of the Canadian Council on Animal Care and the Province of Ontario's Animals for Research Act.

### Mice

C57BL/6 (B6, H-2^b^), BALB/c (H-2^d^) and (B6xBALB/c)F1 (CB6F1, H-2^b/d^) mice were from Jackson Laboratories (Bar Harbor, ME) or Harlan Sprague Dawley (Indianapolis, IN). B6 mice carrying the Fas*^lpr/lpr^* mutation (B6.*lpr*), the FasL*^gld/gld^* mutation (B6.*gld*), Thy1.1-congenic B6 mice (B6. Thy1.1), and IFNγR-targeted B6 and BALB/c mice were from Jackson Laboratories and bred in-house. IFNγ-targeted B6 mice (B6.IFNγ^−/−^) were a gift of Dr. D. Kelvin and bred in-house. B6. Thy1.1.IFNγR^−/−^, B6.*lpr*.IFNγ^−/−^, B6.*lpr*.IFNγR^−/−^, and CB6F1.IFNγR^−/−^ mice were generated by crossing appropriate strains.

### Antibodies and flow cytometry

These mAbs were from eBioscience (San Diego, CA) or BioLegend (San Diego, CA): PE-conjugated anti-IFNγ, anti-CD4, anti-CD8, anti-NK1.1, anti-CD11b, anti-CD11c, anti-CD19, anti-Gr1, Ter119, and anti-γδ TCR; allophycocyanin-conjugated anti-Thy1.1 and anti-CD4; FITC-conjugated anti-TCRβ and anti-IFNγ; biotinylated anti-CD119 (IFNγR) and anti-FasL; purified anti-CD28. Biotinylated antibodies were detected using PE-Cy7-conjugated streptavidin (eBioscience). Anti-CD3 antibody (clone 145-2C11) was prepared from a hybridoma in house. Anti-mouse IFNγ mAb (clone H22) was from R&D Systems (Minneapolis, MN), and IgG isotype control was from eBioscience. Flow cytometry was performed on a Cytomics FC500 (Beckman Coulter, Mississauga ON), an LSR II (Becton Dickinson, Mississauga ON), or an Accuri C6 (Accuri Cytometers, Ann Arbor MI). Dead cells were excluded with propidium iodide (Sigma-Aldrich, Oakville ON), fixable viability dye eFluor450 or 7-aminoactinomycin D (7-AAD, eBioscience).

### Cell purification, BMT and GVHD induction

TCD BM (>99% T cell free) was obtained by treating erythrocyte-free BM cells with anti-Thy1.2 ascites (TIB-107 hybridoma, ATCC, Manassas, VA) and Low-Tox M rabbit complement (Cedarlane Labs, Burlington ON). CD4^+^ T cells (>90% pure) were purified from B6. Thy1.1 spleen and lymph node cells with anti-CD4 microbeads (Miltenyi Biotec, Auburn CA). To obtain DN T cells for BMT, BALB/c, B6.*lpr*, B6.*lpr*.IFNγ^−/−^, or B6.*lpr*.IFNγR^−/−^ mice aged 8-12 weeks were infused with 40×10^6^ CB6F1 splenocytes to activate and expand DN T cells [2], [12]. B6.*lpr* DN T cells were purified by removing CD4^+^, CD8^+^, NK1.1^+^, CD19^+^, CD11b^+^, CD11c^+^, γδTCR^+^, and Ter119^+^ populations with PE-conjugated mAbs and anti-PE microbeads (Miltenyi Biotec; resulting population >99% PE^−^ and 75–90% TCRβ^+^CD4^−^CD8^−^NK1.1^−^ cells; **Fig. S1**). BALB/c DN T cells were enriched by removing B cells by adherence to IgG-coated plates followed by depletion of CD8^+^ and CD4^+^ T cells with anti-CD4 and anti-CD8 hybridoma supernatants (RL172-4 and 3.168, respectively) and Low-Tox M rabbit complement (final preparation up to 50% DN T cells with residual B cells constituting the remainder). Cell number was adjusted based DN T cell purity to ensure that a consistent number of DN T cells were used. Male CB6F1 mice aged 6–10 weeks received 13Gy γ-irradiation (divided doses, >4h apart) in a Gammacell 40 ^137^Cs irradiator (MDS Nordion, Ottawa ON) and were infused with 2×10^6^ TCD BM alone or with 10^6^ CD4^+^ T cells, with/without 2.5–5×10^6^ DN T cells. Survival was monitored daily. Weights and clinical scores [26] were determined 2–3 times weekly. Moribund mice (score >6 or weight loss >25%) were sacrificed. Dead mice were assigned the maximum clinical score of 10. In some experiments, mice were sacrificed 2 weeks post-BMT to obtain tissues.

### Histology and immunohistochemistry

Tissue specimens were snap-frozen and stored at −80°C. Frozen sections were stained with biotinylated anti-Thy1.1 mAb (eBioscience) and horseradish peroxidase-conjugated ultrastreptavidin (ID labs, London ON). Colour was developed with NovaRed (Vector Labs, Burlington ON), with haematoxylin counterstaining. Sections were examined using a Nikon Eclipse E200 microscope (Nikon Canada Inc., Mississauga ON). A blinded observer (C.J.) counted Thy1.1^+^ cells in 10 random high-powered (40× objective) fields. Photomicrographs were acquired using a Leica DM2000 microscope and Infinity 1 camera (Lumenera Corporation, Ottawa ON). In parallel, H&E-stained paraffin sections were examined by a pathologist (O.A.) in blinded fashion to confirm GVHD.

### Suppression assay

CD4^+^ T cells (10^7^ cells/mL) were incubated (10 minutes, 37°C) in PBS containing 1 μM CFSE (Invitrogen, Carlsbad CA), followed by quenching with FBS (Gibco, Carlsbad CA). Cells were washed in α-minimum essential medium with 10% FBS, 50 mM β-mercaptoethanol, 0.1mg/mL penicillin and 0.1 mg/mL streptomycin (CM). 10^5^ CFSE labelled cells were cultured for 5 days with 2×10^5^ irradiated (20Gy) CB6F1 splenocytes and 50 U/mL recombinant human IL-2 (Proleukin, Chiron Corporation, Emeryville CA). Some experiments were performed using plate bound anti-CD3 and soluble anti-CD28 (5 μg/mL) antibodies instead of irradiated CB6F1 splenocytes. Purified DN T cells were added in varying ratios. Propidium iodide-negative responder cells were identified with anti-CD4 and/or anti-Thy1.1 antibodies and CFSE and subjected to flow cytometry. Percent suppression was calculated using the formula: [(%CFSE^hi^ (DN+CD4^+^) – %CFSE^hi^ (CD4^+^ only))/(100-%CFSE^hi^ (CD4^+^ only))] ×100%. In some experiments, anti-IFNγ mAb (2.5 μg/mL) or isotype control were added. In others, 7-AAD staining and CFSE dilution were jointly examined. Cell death in the presence of B6.*lpr* DN T cells was compared to cell death in their absence by calculating fold change, defined as (%7-AAD^+^ (DN+CD4^+^))/(%7-AAD^+^ (CD4^+^ only)).

### IFNγ staining

Two million splenocytes were cultured for 4 h in 2 mL CM containing GolgiStop (monensin, 1.5 μL/mL, BD Biosciences, Mississauga ON), with/without 50 ng/mL PMA and 500 ng/mL ionomycin (Sigma-Aldrich). Cells were stained for TCRβ, CD4, CD8, and NK1.1. IFNγ staining was performed with an intracellular staining kit (eBioscience).

### Cytotoxicity assay

The JAM assay [44] was used to quantitate cytotoxicity as described previously [12]. Briefly, 2×10^6^ B6 CD4^+^ T cells were added to 10×10^6^ irradiated CB6F1 splenocytes and 50 U/mL IL-2 in 2 mL CM. After 4 days, 10 μCi/mL ^3^H-thymidine (Perkin-Elmer, Woodbridge ON) was added for 18 h. In parallel, 5×10^6^ B6.*lpr*, B6.*lpr*.IFNγ^−/−^, or B6.*lpr*.IFNγR^−/−^ DN T cells were activated in similar fashion without ^3^H-thymidine. Then, 10^4^ ^3^H-thymidine-labelled CD4^+^ T cells were co-cultured in duplicate or triplicate 200 μL cultures with 5×10^4^ fresh irradiated CB6F1 splenocytes and B6.*lpr* DN T cells in varying ratios. The cpm of retained DNA, measured on a TopCount NXT (Perkin-Elmer), was used as an index of cell survival. At each DN:CD4^+^ ratio, the percent CD4^+^ killing was determined using the formula: [(cpm(CD4^+^ only) – cpm(DN+CD4^+^))/cpm(CD4^+^ alone)] ×100%.

### IFNγ ELISA

Serum samples were assayed in duplicate for IFNγ content using a Quantikine Mouse IFNγ ELISA kit (R&D Systems), according to the manufacturer's instructions.

### DN T cell FasL expression

Five million spleen and lymph node cells from B6.*lpr*, B6.*lpr*.IFNγ^−/−^, and B6.*lpr*.IFNγR^−/−^ mice were cultured in 2 mL CM in plates coated with anti-CD3 mAb, along with 50 U/mL IL-2 and 5 μg/mL soluble anti-CD28 mAb with/without 100 ng/mL recombinant mouse IFNγ (R&D Systems). In some cases anti-IFNγ antibody (2.5 μg/mL) or isotype control were included; in others, 50 µM TAPI-1 [45] (Peptides International, Louisville KY) or DMSO vehicle were added for the final 18h of culture. After 48 h, cells were stained for TCRβ, CD4, CD8, and NK1.1. Half of the cells were stained for surface FasL expression and fixed; the remainder were stained intracellularly for FasL.

### Data Analysis

Flow cytometry data were analyzed and presented with FlowJo 7 (Treestar, Ashland OR). Graphical presentation and statistical analysis of data were performed with Prism 5.0 (GraphPad, La Jolla CA). Survival analysis was performed using the log-rank test. Variance between two groups was assessed with either Student's t test (in some cases with Welch's correction for unequal variances) or the Mann-Whitney test; three or more groups were analyzed by ANOVA, in some cases with Bonferroni's post-test to compare groups, or the Kruskal-Wallis test in the case of non-parametric data. A p value of less than 0.05 was considered statistically significant. Error bars in all graphs represent standard deviations.

## Supporting Information

Figure S1 **Purification of B6.lpr DN T cells.** Pooled spleen and lymph node cells from B6.*lpr* mice were incubated with PE-conjugated antibodies to CD4, CD8, NK1.1, CD11b, CD11c, Ter119, γδTCR, and CD19, washed, and incubated with anti-PE microbeads. PE^+^ cells were then removed using LD columns. Aliquots of the pre-column population (left panels) and the negative fraction (right panels) were stained with FITC-conjugated TCRβ antibody and analyzed by flow cytometry. Two examples of DN T cell purification from B6.*lpr* mice are shown.
(TIF)

Figure S2 **Matrix metalloproteinase inhibition results in a slight increase in FasL expression on B6.lpr DN T cells, regardless of their ability to secrete and respond to IFNγ.** Spleen and lymph node cells from B6.*lpr*, B6.*lpr*.IFNγ^−/−^, and B6.*lpr*.IFNγR^−/−^ mice were activated as in Fig. 6A with plate-bound anti-CD3, soluble anti-CD28, and IL-2 for 48h. During the final 18h of culture, either 50 μM TAPI-1 (dotted lines) or its DMSO vehicle (solid lines) was added to the cultures. Cells were then stained for TCRβ, CD4, CD8, NK1.1, and FasL prior to fixation and analysis by flow cytometry. Each histogram shows data from one mouse per genotype; data are from one of two experiments each with 2 mice per genotype.
(TIF)

Figure S3 **Fas expression by proliferating CD4^+^ T cells determines their susceptibility to killing by activated DN T cells.** CFSE-labelled B6.*lpr* (Fas^LPR/LPR^) or B6. Thy1.1 (Fas^+/+^) CD4^+^ T cells were cultured with irradiated CB6F1 splenocytes and IL-2, without or with B6.*lpr* DN T cells for 5 days. These data are from the same experiment as in Fig. 7B. Responder cells, identified by CD4 (or Thy1.1) and CFSE, were stained with 7-AAD and analyzed by flow cytometry. Histograms at left show 7-AAD staining of divided (CFSE-diluted) cells for Fas^+/+^ Thy1.1 cells (top row) and Fas^LPR/LPR^ cells (bottom row). Numbers inside histograms reflect the percentage of 7-AAD^+^ cells within the gate. The fold increase in dead CD4^+^ T cells, defined as the percent 7-AAD^+^ within the divided population divided by the percent 7-AAD^+^ within the undivided population, is shown in the graph at right. Data are derived from duplicate wells in one of two experiments with similar results. Two-way ANOVA p<0.0001; Bonferroni post test ***p<0.001; **p<0.01; *p<0.05.
(TIF)
