# Supplementary figures and images for: Autocrine IFNγ Controls the Regulatory Function of Lymphoproliferative Double Negative T Cells
Source: PLoS One. 2012 Oct 15;7(10):e47732. doi: 10.1371/journal.pone.0047732 (PMC3471870; doi:10.1371/journal.pone.0047732)

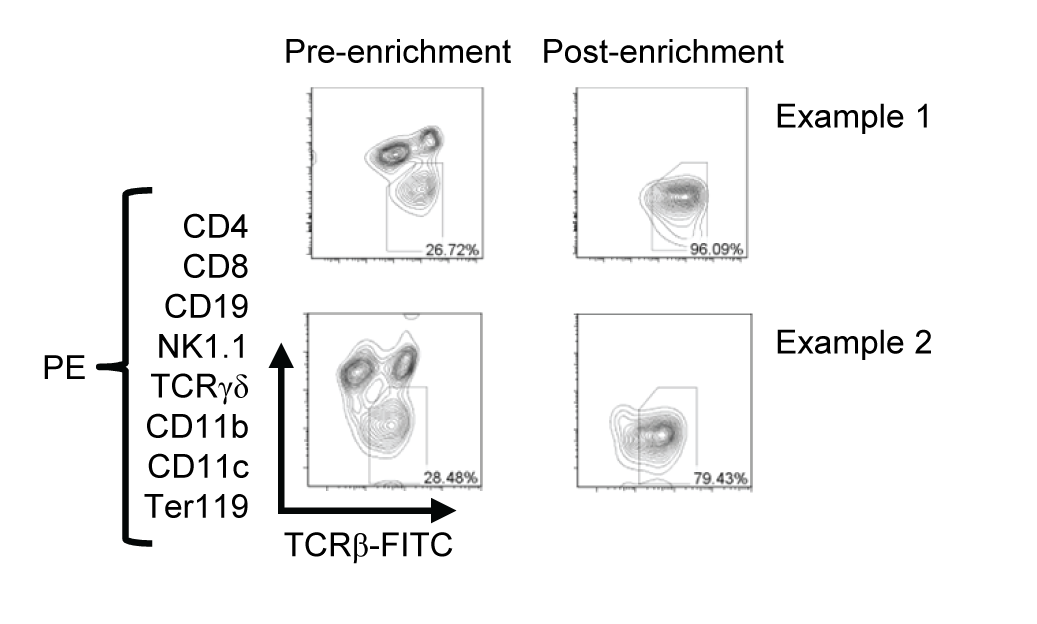

Supplement: Figure S1 — Purification of B6.lpr DN T cells. Pooled spleen and lymph node cells from B6.lpr mice were incubated with PE-conjugated antibodies to CD4, CD8, NK1.1, CD11b, CD11c, Ter119, γδTCR, and CD19, washed, and incubated with anti-PE microbeads. PE+ cells were then removed using LD columns. Aliquots of the pre-column population (left panels) and the negative fraction (right panels) were stained with FITC-conjugated TCRβ antibody and analyzed by flow cytometry. Two examples of DN T cell purification from B6.lpr mice are shown. (TIF) [file pone.0047732.s001.tif]

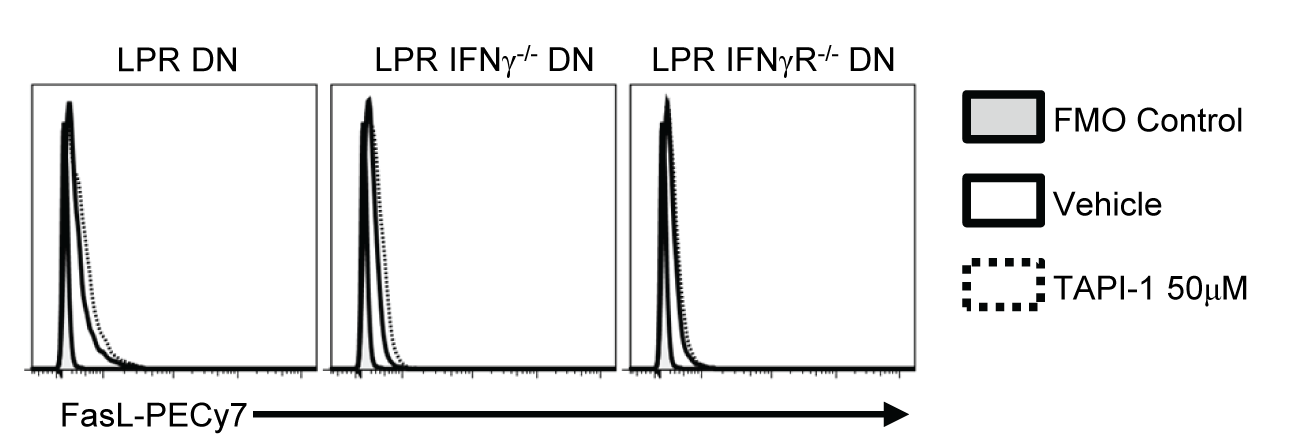

Supplement: Figure S2 — Matrix metalloproteinase inhibition results in a slight increase in FasL expression on B6.lpr DN T cells, regardless of their ability to secrete and respond to IFNγ. Spleen and lymph node cells from B6.lpr, B6.lpr.IFNγ−/−, and B6.lpr.IFNγR−/− mice were activated as in Fig. 6A with plate-bound anti-CD3, soluble anti-CD28, and IL-2 for 48h. During the final 18h of culture, either 50 μM TAPI-1 (dotted lines) or its DMSO vehicle (solid lines) was added to the cultures. Cells were then stained for TCRβ, CD4, CD8, NK1.1, and FasL prior to fixation and analysis by flow cytometry. Each histogram shows data from one mouse per genotype; data are from one of two experiments each with 2 mice per genotype. (TIF) [file pone.0047732.s002.tif]

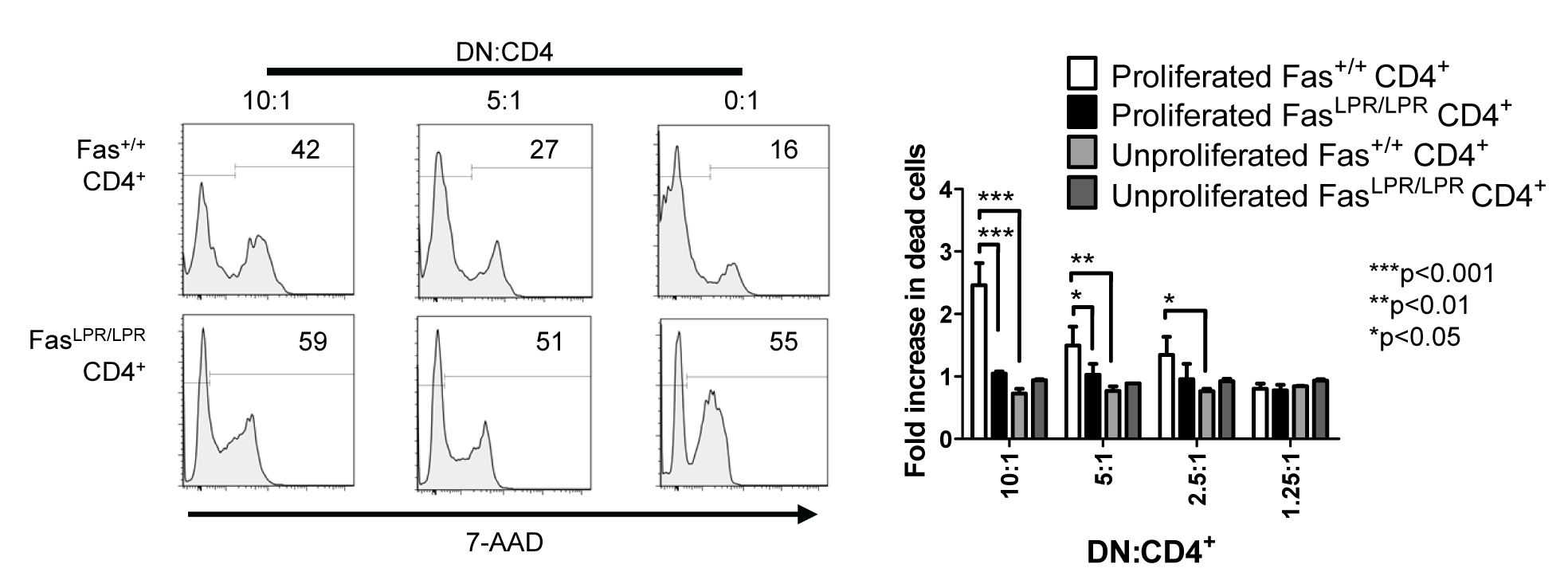

Supplement: Figure S3 — Fas expression by proliferating CD4+ T cells determines their susceptibility to killing by activated DN T cells. CFSE-labelled B6.lpr (FasLPR/LPR) or B6. Thy1.1 (Fas+/+) CD4+ T cells were cultured with irradiated CB6F1 splenocytes and IL-2, without or with B6.lpr DN T cells for 5 days. These data are from the same experiment as in Fig. 7B. Responder cells, identified by CD4 (or Thy1.1) and CFSE, were stained with 7-AAD and analyzed by flow cytometry. Histograms at left show 7-AAD staining of divided (CFSE-diluted) cells for Fas+/+ Thy1.1 cells (top row) and FasLPR/LPR cells (bottom row). Numbers inside histograms reflect the percentage of 7-AAD+ cells within the gate. The fold increase in dead CD4+ T cells, defined as the percent 7-AAD+ within the divided population divided by the percent 7-AAD+ within the undivided population, is shown in the graph at right. Data are derived from duplicate wells in one of two experiments with similar results. Two-way ANOVA p<0.0001; Bonferroni post test ***p<0.001; **p<0.01; *p<0.05. (TIF) [file pone.0047732.s003.tif]
